# Enhancing Micro-Raman Spectroscopy: A Variable Spectral Resolution Instrument Using Zoom Lens Technology

**DOI:** 10.3390/s24134284

**Published:** 2024-07-01

**Authors:** Ivan Pavić, Nediljko Kaštelan, Arkadiusz Adamczyk, Mile Ivanda

**Affiliations:** 1 Department for Marine Electrical Engineering and Information Technologies, Faculty of Maritime Studies, Ruđera Boškovića 37, 21000 Split, Croatia; ipavic@pfst.hr (I.P.); nkastelan@pfst.hr (N.K.); 2 Faculty of Mechanical and Electrical Engineering, Polish Naval Academy, ul. Smidowicza 69, 81-127 Gdynia, Poland; a.adamczyk@amw.gdynia.pl; 3 Laboratory for Molecular Physics and Synthesis of New Materials, Ruđer Bošković Institute, Bijenička Cesta 54, 10000 Zagreb, Croatia

**Keywords:** Raman spectroscopy, high-resolution Raman spectrometer, zoom lens

## Abstract

Raman spectroscopy is a powerful analytical technique based on the inelastic scattering of photons. Conventional macro-Raman spectrometers are suitable for mass analysis but often lack the spatial resolution required to accurately examine microscopic regions of interest. For this reason, the development of micro-Raman spectrometers has been driven forward. However, even with micro-Raman spectrometers, high resolution is required to gain better insight into materials that provide low-intensity Raman signals. Here, we show the development of a micro-Raman spectrometer with implemented zoom lens technology. We found that by replacing a second collimating mirror in the monochromator with a zoom lens, the spectral resolution could be continuously adjusted at different zoom factors, i.e., high resolution was achieved at a higher zoom factor and lower spectral resolution was achieved at a lower zoom factor. A quantitative analysis of a micro-Raman spectrometer was performed and the spectral resolution was analysed by FWHM using the Gaussian fit. Validation was also performed by comparing the results obtained with those of a high-grade laboratory Raman spectrometer. A quantitative analysis was also performed using the ANOVA method and by assessing the signal-to-noise ratio between the two systems.

## 1. Introduction

The Raman effect is a phenomenon that describes the interaction of light with matter, which leads to light scattering due to the vibrational state of the molecules. This scattering differs depending on the vibrational modes of the molecules and is, therefore, considered a “fingerprint” for molecule detection [1,2]. The Raman effect is of great use in various scientific fields, including chemistry, physics, biology and materials science [3,4,5,6,7,8]. In addition, two types of scattering can occur: Stokes scattering, in which the scattered light has a longer wavelength (lower energy) than the incident light, and anti-Stokes scattering, in which the wavelength is shorter (higher energy) than the incident light [9,10]. Two types of Raman spectrometers, namely, macro-Raman spectrometers and micro-Raman spectrometers [11,12,13], are usually used in standard laboratory work. The main difference between these spectrometers is the spatial resolution and the size of the sample to be analysed. For analysing larger sample areas, macro-Raman spectrometers are used, while micro-Raman spectrometers offer high spatial resolution so that smaller sample areas up to one micrometre can be analysed in more detail [11,14,15,16,17]. In addition, the design of a macro-Raman spectrometer is characterised by a simple optical configuration and larger collection optics to capture larger sample areas. In contrast, a micro-Raman spectrometer uses complex optical configurations, including microscope objectives, to focus the laser beam on a small spot on a sample and efficiently collect the scattered light from that small area, enabling high-spatial-resolution analysis [18,19,20]. Another approach for carrying out detailed molecular analyses is the development of fibre-optic Raman probes. These probes involve the integration of multiple lenses to optimise the collimation, dispersion and focusing of the light. It should be noted that fibre-optic Raman probes allow for remote measurements in hazardous or difficult-to-access areas, such as in vivo medical or industrial environments [21,22,23,24]. However, the development of such a probe is challenging and has already been investigated by several authors [21,25,26]. Therefore, this article addresses a standard approach for the development of micro-Raman spectrometers utilising variable spectral resolution. Furthermore, the spectral resolution of both types of spectrometer depends on the following factors: diffraction grating densities, where a higher number of grows per millimetre results in finer spectral resolution; the entrance slit width of the monochromator, which controls the amount of light entering the monochromator; the focal length of collimating optics within monochromators, where a longer optical focus leads to higher spectral resolution; and detector characteristics like pixel size, density and sensitivity, regardless of whether it is a charge-coupled device (CCD), a combined metal-oxide-semiconductor (CMOS) detector or an electron-multiplying CCD (EMCCD) [27].

In our earlier research [28], we tested the use of a zoom lens instead of a second collimating mirror and developed a new concept for a simple macro-Raman spectrometer with variable spectral resolution. A zoom lens with a maximal focus magnification of 6× was used. In our experiment, the spectral resolution was calculated using Gaussian fit for each Stokes peak of the Raman spectrum of ZrO_2_ at different zoom factors. The results showed that the spectral resolution was 18.78 cm^−1^ for the maximum zoom factor (ZF), i.e., 6× magnification, and 78.42 cm^−1^ for the minimum ZF of 1× for the characteristic line of ZrO_2_ at 483.5 cm^−1^. The observed increase in spectral resolution by 4.17 times at 6× magnification was a little bit worse than expected (6 times) due to the number of different aberrations in the collimating optical system.

By using the concept of a zoom lens in Raman spectroscopy, in this study, we further extended our previous work with the development of a complete prototype micro-Raman spectrometer with variable spectral resolution. Its construction, with all the details on the components and materials used for the test measurements, is described and explained here. We compared the prototype with a reference system, i.e., the high-quality laboratory Raman spectrometer JY-T6400. A quantitative analysis was then carried out by comparing the full-width half maximum (FWHM) of the specific spectral lines and statistically analysing the variance (ANOVA) of the spectra and signal-to-noise ratio (SNR) for both the prototype and the reference system.

## 2. Materials and Methods

In this chapter, the design of the micro-Raman spectrometer prototype with variable spectral resolution, together with its assembly and the materials used for the measurements, are described.

### 2.1. Raman Spectrometer Components

Every Raman spectrometer consists of three main parts: the source of light for the excitation, the unit for the spectral dispersion and the light detection unit. These units compromise a number of mechanical and optical components such as filters, focusing lenses, slits, gratings, mirrors, etc. All these components are listed in Table 1. It should be noted that most of the components were off-the-shelf, except for the CCD detector and the electronic part for the spectrometer control.

**Figure 1:**
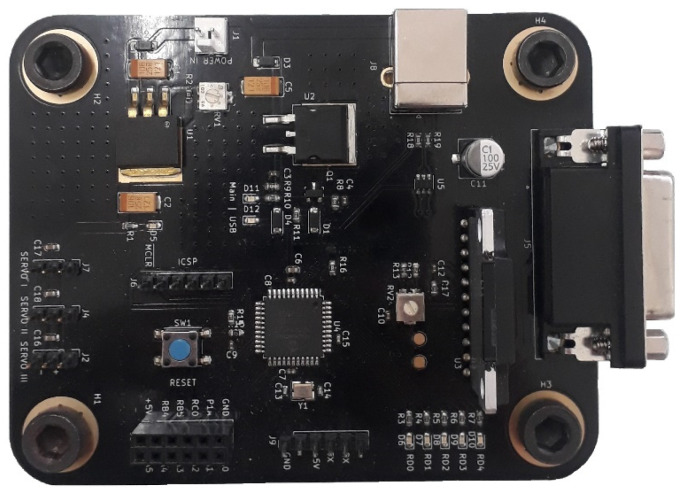
Completed two-layer PCB for electronic hardware used in micro-Raman spectrometer with variable spectral resolution.

Furthermore, additional electronic hardware (Printed Circuit Board–PCB), shown in Figure 1, was designed to control the angle of diffracting grating inside the monochromator unit, the zoom factor of the zoom lens and the external shutter.

It should be noted that the Trius PRO-694 monochromatic CCD optical sensor was chosen for its ability to capture high-resolution images with 2750 × 2200 pixels. The pixel size was 4.54 μm, resulting in an active area of 12.5 × 10 mm, with a maximum binning of 16 × 16 pixels. A 16-bit analogue-to-digital converter to process the information for each pixel and internal Peltier cooling for noise reduction were also used.

### 2.2. Micro-Raman Spectrometer Prototype Assembly

**Figure 2:**
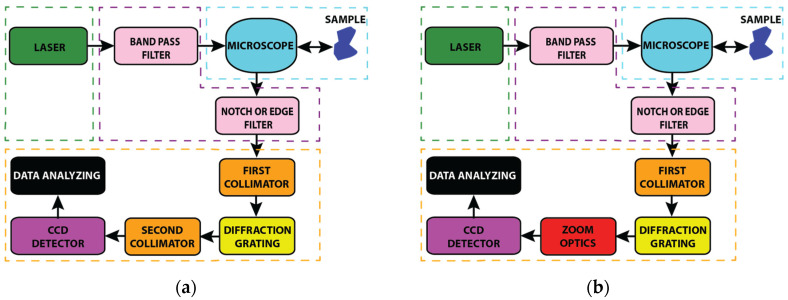
Schematic diagram of micro-Raman spectrometer prototype: (**a**) standard approach and (**b**) implementation of a zoom optics instead of second collimator to achieve spectrometer with variable spectral resolution.

After the components were selected, the micro-Raman spectrometer prototype was assembled as shown in Figure 2.

**Figure 3:**
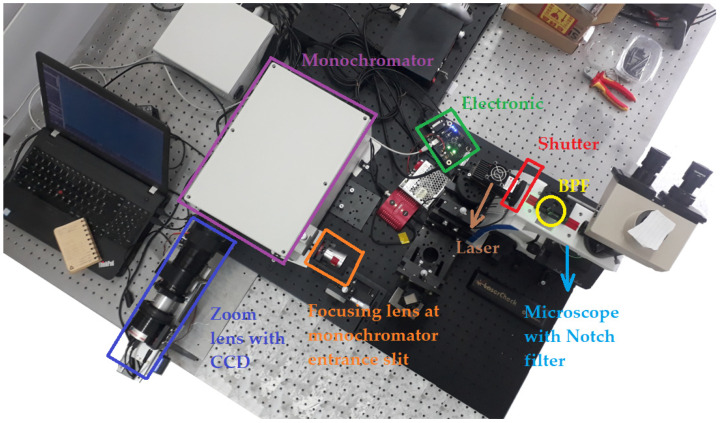
Fully assembled micro-Raman spectrometer with variable spectral resolution.

To achieve the micro-Raman spectrometer prototype with variable spectral resolution, the second collimating/focusing mirror was replaced with a zoom lens (Figure 2b). Assembly began with the precise alignment of the laser beam in the microscope entrance using the stage with X-Y-Z micrometre screws. In order to maintain the laser beam on the optical axes, the laser power was set to 100 mW for a consistent comparison. The laser power was measured using a Coherent Laser Check Power Meter. After aligning the laser, a 30:70 beam splitter was placed at a 45° angle to focus the laser beam onto the sample at the centre of the view field. The measured laser power on the sample was 21 mW, indicating good collimation. A reflection of the laser beam by a polished silicon surface helped to direct the parallel beam back to the microscope exit by using a 50× confocal microscope objective. The power measured at the output was 12.5 mW. By using mirrors, the laser beam (including the Raman signal) was led to the focusing lens at the front of the first slit of the monochromator. The size of the focused spot that entered the monochromator was 35 microns. Further, adjustments were made inside the spectrometer to align the beam to the centre of the grating. After adjustment, the notch filter was placed on a parallel beam within the tube of the microscope. A zoom lens connected to a CCD was installed together with electronic hardware and the necessary filters so that the micro-Raman spectrometer prototype with variable spectral resolution could be fully assembled, as shown in Figure 3.

After assembly, it was necessary to calibrate the x positions of the CCD pixels into the Raman shift or wavenumbers. Calibration began by setting the zoom factor to 1× and using lasers with wavelengths of 532 nm and 785 nm. First, a 532 nm laser was used and the grating was rotated so that the laser spot was on the left side of a CCD, and the pixel position of the spot was recorded. Next, the 532 nm laser was replaced by a 785 nm laser and the pixel position was also recorded. The distance between the spots resulted in a spectral length of about 8 cm−1 per pixel at the lowest zoom factor. This calibration process was repeated for different zoom factors, resulting in approximately 0.44 cm^−1^ per pixel at a maximum zoom factor of 18×. The conversion between cm^−1^ and wavenumber was performed according to a predefined formula [27,28].

Furthermore, the micro-Raman spectrometer prototype had the following parameters: 306 mm focal length at zoom factor 18×, slit width of 50 μm and diffraction grating of 1100 grooves/mm.

### 2.3. Materials

The Raman spectra of different materials, shown in Table 2, were recorded. To test the effects of variable spectral resolution, samples of silicon (Si) and sulphur (S) were used because they provide strong Raman bands at known positions. Furthermore, for the quantitave analysis of spectral resolution, together with Si and S, a powder titanium dioxide TiO_2_-anatase/P25 and TiO_2_/rutile (anatase annealed at 800° for 1 h) was used. It has to be noted that all samples were placed on a separate microscopic glass under the microscope. For each sample, the laser beam was manually focused on the sample.

### 2.4. Quantitave Analysis

First, the Raman spectra were receded on Si and S, varying the zoom factor (ZF). Next, for different ZFs, a spectral resolution was analytically calculated using Gaussian function fit for Raman bands to estimate the FWHM [29,30]. Furthermore, the quality of the Raman spectra was estimated by analyzing the signal-to-noise ratio (SNR) and then compared with the reference Raman spectrometer system under similar conditions. Then, the obtained results from a micro-Raman spectrometer prototype were validated using statistical metrics and the analysis of variance (ANOVA) method [31,32,33]. For reference, the Raman spectrometer Horiba JY-T64000 was used under the following specifications: liquid nitrogen-cooled CCD (Cryogenic, Symphony I, 1024 × 256 pixels, pixel size 26 × 26 μm), 640 mm focal length monochromator, slit width of 50 μm and diffraction grating of 1800 grooves/mm.

## 3. Results

This chapter discusses the results obtained from the micro-Raman spectrometer prototype and the reference system (JY-T64000).

### 3.1. Raman Spectra

**Figure 4:**
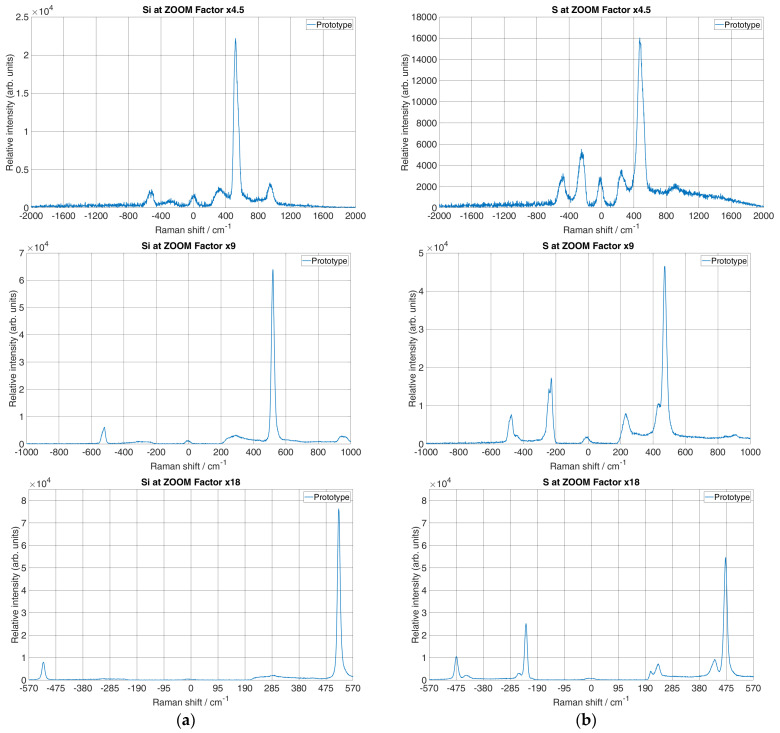
Acquired Raman spectrum with a prototype spectrometer for (**a**) Si with ZF 4.5×, 9× and 18× and (**b**) S with ZF 4.5×, 9× and 18×.

The micro-Raman spectrometer prototype was set to 1 s exposure time and the laser power on the sample was set to 15 mW. The Raman spectra of the Si and S acquired with the prototype at the different ZFs are shown in Figure 4, where the x-axis denotes Raman shift with units in cm^−1^, whereas the y-axis represents relative intensity with arbitrary units. It has to be noted that both Si and S ZF measurements were set to a value where Raman lines were visible and recognisable. Therefore, for both samples, the initial ZF was set to 4.5× magnification. The grating was rotated so that the laser line was in the middle of the x-axis.

The spectral interval over the entire CCD region at a ZF of 4.5× ranged from 480 to 596 nm. At this ZF, the spectrum of Si showed a line of the transversal optical (TO) vibrational mode at 520 cm^−1^ and a second harmonic line at 900 to 1000 cm^−1^ as well as the corresponding anti-Stokes spectrum. Furthermore, the S spectrum, at the same ZF, showed a strong optical phonon line at 472.8 cm^−1^. However, it was necessary to increase the resolution by adjusting the ZF to see more spectral components. Next, the ZF was further increased by a factor of 2, i.e., from the initial value to a ZF of 9×. For this ZF, Si showed Stokes and anti-Stokes peaks, which become narrower due to the increased spectral resolution. A similar observation for Stokes and anti-Stokes peaks was observed for S. It has to be noted that for this ZF, the spectral interval over the entire CCD region ranged from 505 to 562 nm in wavelength. Furthermore, if the ZF was set to the maximum value of 18×, it could be observed that the spectral interval across the CCD region became even narrower, measuring 516 to 549 nm in wavelength. Here, the spectral lines for both Si and S were more distinct than at lower ZFs. However, at this ZF, it could be seen that the TO second harmonic mode of Si was out of the range of detection. The total spectral interval over the CCD region was reduced by increasing the ZF, while the spectral resolution increased which was directly evident by line narrowing.

For a correct comparison, it was necessary to achieve similar spectral resolutions of the micro-Raman spectrometer prototype and the reference system. For this purpose, firstly the reciprocal linear dispersion was calculated as in [28] for both systems. By substituting the value of the focal length *f* = 306 mm (with Barlow 2× magnification) for the maximal ZF, the order of the diffraction *n* = 1, the distance between ruling *d* = 1/1100 mm and the reciprocal linear dispersion for the first-order diffraction *D*^−1^ = 2.971 nm/mm. Furthermore, for the micro-Raman spectrometer prototype, with a pixel size of 4.54 μm, the active region of the CCD was 12.5 mm in width. Therefore, the per-pixel width was 0.0135 nm/pixel. The spectral dispersion at the position of the Raman band for Si at 520 cm^−1^ and S at 472.77 cm^−1^ was 0.46 cm^−1^/pixel. Furthermore, for the reference system, with *f* = 640 mm, *n* = 1 and *d* = 1/1800 mm, the reciprocal linear dispersion was 0.868 nm/mm. The per-pixel width was 0.0226 nm/pixel, which gave a dispersion of 0.776 cm^−1^/pixel, a 1.68 times higher value. Therefore, a binning factor of 1× was used during recording by the micro-Raman spectrometer prototype.

Spectral resolution depends on the input slit of the spectrometer. Therefore, the input slit width for the prototype was set to 35 μm. Furthermore, the collimating optics of the micro-Raman spectrometer prototype were f_co1_ = 230 mm and, for the maximal ZF of 18×, the imaging optics were f_co2_ = 306 mm. Therefore, the total magnification of the monochromator was f_co2_/f_co1_ = 1.33, which gave an image size of 46.55 μm at the CCD. From the Nyquist limit, three times the image size over the CCD was used to determine whether the CCD could differentiate between the two neighbouring spectral lines. Therefore, the CCD could differentiate between two adjacent spectral lines when their peaks were separated by 139.65 μm on the CCD, which, spectrally, was 14.15 cm^−1^. This can be seen from Figure 4b, with an 18× ZF at a position of about −240 cm^−1^, where two peaks that appeared with a 25 cm^−1^ difference between them were completely resolved, while with a 4.5× ZF, the same Raman bands were shown as one peak.

### 3.2. Spectral Resolution

**Figure 5:**
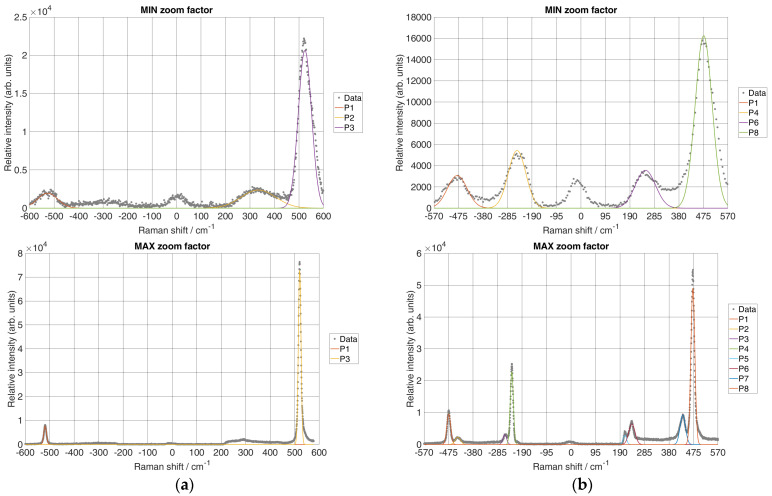
Calculation of the spectral resolution of the micro-Raman spectrometer prototype with minimal and maximal ZFs: (**a**) Raman spectra for Si using the Gaussian fit for each peak and (**b**) Raman spectra for S using the Gaussian fit for each peak. Note that the peak at location 0 cm^−1^ was the peak of the excitation source. Therefore, the Gaussian fit was not calculated for this peak.

The spectral resolution of a prototype was calculated by measuring the linewidth at the minimum and maximum ZFs, i.e., at 4.5× and 18× for the Si Raman line at 520 cm^−1^ (Figure 4a) and the S Raman line at 472 cm^−1^ (Figure 4b). Therefore, the Gaussian function was used to fit the Raman lines to estimate the FWHM at different ZFs, as in [28]. Figure 5 shows the results of the fit for both materials.

As shown in Figure 5a, the measured linewidth for the 520 cm^−1^ Raman band of Si resulted in 63.61 cm^−1^ with the minimum ZF and 12.18 cm^−1^ with the maximum ZF. Furthermore, as shown in Figure 5b, the measured linewidth for the 472 cm^−1^ Raman band resulted in 76.97 cm^−1^ with the minimum ZF and 18.25 cm^−1^ with the maximum ZF. The results of the Gaussian fitting parameters for Si and S with their respective peaks at the minimum and maximum ZFs, together with their FWHM values, are shown in Table 3.

From the calculated linewidth of the characteristic peak at 520.24 cm^−1^ for Si and the characteristic peak at 472.44 cm^−1^ for S, it can be seen that the spectral resolution at the maximum ZF increased significantly, i.e., the spectral resolution became around four times larger than the spectral resolution for the same peak at the minimum ZF.

**Figure 6:**
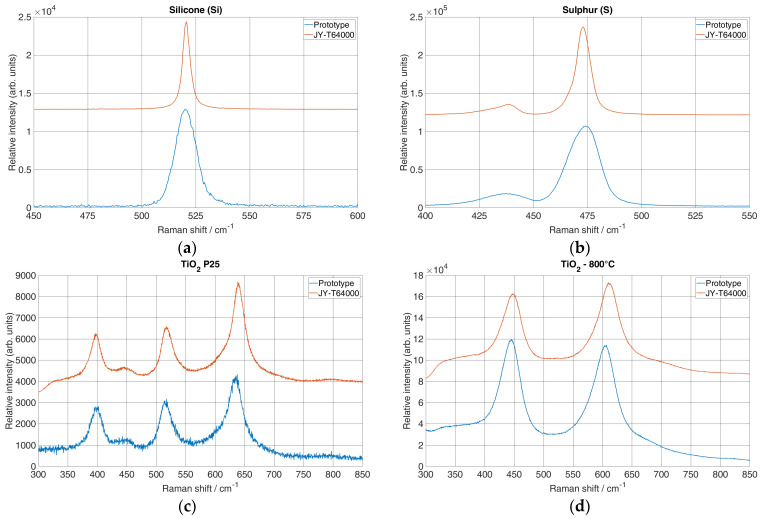
Spectra obtained by reference system and proposed system used for difference analysis between the two systems: (**a**) Raman spectra for Si, (**b**) Raman spectra for S, (**c**) Raman spectra for TiO_2_ P25 and (**d**) Raman spectra for TiO_2_/800°, 1 h.

With an image size of 46.55 μm at the CCD, a pixel size of 4.54 μm and 0.46 cm^−1^/pixel, the true Raman bandwidth was $\Delta {\tilde{v}}_{slit}$ = 4.715 cm^−1^. Next, for the true Raman line of Si for the characteristic peak at 520.24 cm^−1^, measured with the reference $\Delta {\tilde{v}}_{line}$ = 4 cm^−1^ (Figure 6a) and fitted FWHM = 12.81 cm^−1^ (Table 2) (maximum ZF), it was found that $\Delta {\tilde{v}}_{resolution}$ = 11.13 cm^−1^. Also, for the same peak and its fitted FWHM = 63.61 cm^−1^ (Table 2) (minimum ZF), it was found that $\Delta {\tilde{v}}_{resolution}$ = 63.29 cm^−1^. Next, for S and the characteristic peak at 472.44 cm^−1^, the true Raman line measured by the reference system was $\Delta {\tilde{v}}_{line}$ = 9 cm^−1^ (Figure 6b) and its fitted FWHM = 18.25 cm^−1^ (Table 2) (maximum ZF), and it was found that $\Delta {\tilde{v}}_{resolution}$ = 15.09 cm^−1^. For the same peak and fitted FWHM = 76.97 cm^−1^ (Table 2) (minimum ZF), it was found that $\Delta {\tilde{v}}_{resolution}$ = 76.28 cm^−1^. It can be seen that in both scenarios for Si and S, the difference between the minimum and maximum ZFs was more than four times the spectral resolution. Since the initial ZF = 4.5 was not exact, i.e., there were slight deviations due to the motorised positioning of the ZF on the zoom lens, the spectral resolution changed approximately the same as the ZF since the difference between the minimum and maximum ZFs was 4 times.

### 3.3. Statistical Analysis

To evaluate the collected data between the two systems, a comparison of the spectra was calculated using ANOVA analysis. Figure 6 shows the spectra of the prototype and the reference system for the analysis.

The spectral intervals were selected on the basis of the prominent Raman lines specific to each material: 450 to 600 cm^−1^ for Si, 400 to 550 cm^−1^ for S and 350 to 800 cm^−1^ for TiO_2_ P25 and TiO_2_-800°. An ANOVA table (Table 4) was created for each material using Microsoft Excel 2016 to show the sum of squares (*SS*), degrees of freedom (*df*), mean square values (*MS*), *p*-values and calculated *F* and *F*_critical_ values. A significance level alpha of 0.05 was used throughout. For the null hypothesis, it was assumed that there was no significant difference between the data sets, which meant that the *F*-value needed to be less than the *F*_critical_ value and the *p*-value needed to be greater than the alpha. If the *F*-value exceeded the *F*_critical_ value and the *p*-value was less than the alpha, the null hypothesis was rejected in favour of the alternative. In Table 4, the calculated *F*, *F*_critical_ and *p*-values show no statistical differences for all the compared materials (*F* < *F*_critical_ and *p* > 0.05), so the null hypothesis was not rejected.

### 3.4. Signal-to-Noise Ratio

To measure the quality of a peak compared to the background noise of the micro-Raman spectrometer prototype, the signal-to-noise ratio (SNR) was estimated from experimental spectra and then compared to the reference system. The estimation was performed by applying Savitzky–Golay (SG) smoothing [34,35,36] to the raw data recorded by both systems, with the third-order polynomial and the window frame to 11. The filtered spectra were then subtracted from the experimental spectra to obtain a noise spectrum. The standard deviation was calculated from the noise spectrum. The SNR was roughly estimated using Equation (1) by the ratio between the maximum peak intensity (*S*) and the standard deviation of the background noise (*σ_b_*) [37].

(1) $$SNR=\frac{S}{\sigma_{b}}$$

**Figure 7:**
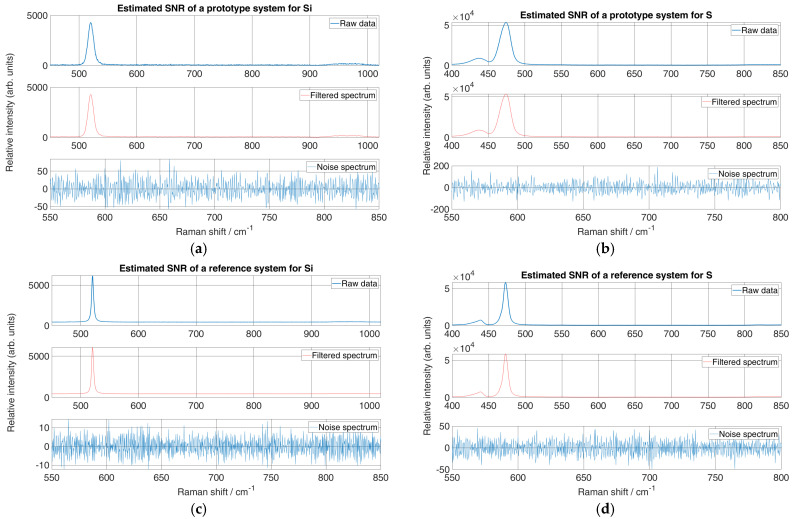
SNR estimation of (**a**) Si Raman spectra from micro-Raman spectrometer prototype, (**b**) S Raman spectra from micro-Raman spectrometer prototype, (**c**) Si Raman spectra from reference system and (**d**) S Raman spectra from reference spectrometer.

It should be noted that the intensity of the reference system was calculated as the average of the y-axis pixels for each pixel of the x-axis, while the intensity of the prototype was calculated as the sum of the y-axis pixels for each pixel of the x-axis. Therefore, to evaluate the SNR difference between the two systems, the intensity of the prototype of the micro-Raman spectrometer was calculated in the same way as that of the reference system. The filtered spectrum with SG smoothing and generated background noise is shown in Figure 7.

The estimated SNR (Table 5) showed that the reference system performed better in terms of peak visibility compared to background noise. Furthermore, the background noise level was about 10 counts above the y-axis for Si and 37 counts above for S. However, the background noise level of the prototype system was higher and was 22 counts above the y-axis for Si and 129.4 counts above for S. This may have been due to the liquid nitrogen-cooled CCD of the reference system and it being housed in a dark room, while the CCD used for the prototype was cooled only with the implemented Peltier cooling and reached a maximum of −20 °C. Therefore, as expected, the estimated SNR of the reference system was at least twice as high as that of the prototype for Si and about three times as high as that for S. Nevertheless, the background noise level of the prototype was consistent when recording spectra of different materials.

## 4. Conclusions

Based on previous research in which a macro-Raman spectrometer was built using a zoom lens instead of a second collimating mirror in the monochromator, a micro-Raman spectrometer prototype was developed in this work. Furthermore, a higher spectral resolution was achieved by using a zoom lens with a maximum magnification of 18× and a high-resolution CCD. Additional electronic hardware was developed to achieve partial autonomy, i.e., grating control, zoom factor adjustment and shutter control.

To test the performance, the prototype micro-Raman spectrometer was compared with the reference spectrometer JY-T64000. The spectra were analysed using the FWHM value estimated by Gaussian fit, the statistical ANOVA method and the signal-to-noise ratio. The results showed that the spectral resolution increased proportionally to the zoom factor, and a fourfold spectral resolution was achieved between the minimum and maximum ZFs, corresponding to a minimum zoom lens with a magnification of 4.5× and a maximum zoom of 18×. In addition, the results of a statistical analysis showed no significant deviation between the prototype micro-Raman spectrometer and the reference spectrometer, further confirming the results obtained with the prototype. However, the signal-to-noise ratio regarding the background noise level of the prototype was found to be at least twice that of the reference spectrometer due to the fact that the reference spectrometer was housed in a dark room and used a liquid nitrogen-cooled CCD sensor.

Although there are commercial spectrometers with dual-mode (low and high resolution) acquisition capabilities, the proposed system offers a distinct advantage as it provides variable spectral size resolution with a single grating. Furthermore, this can be advantageous for the rapid acquisition of different spectral regions, i.e., a minimum ZF can be used for broader spectra and a maximum ZF for narrower spectral regions. Nevertheless, some notes should be considered for future work. First, to increase the spectral resolution, a zoom lens with a higher ZF can be used, increasing the focal length even further. Second, to reduce the ambient light entering the spectrometer, an optical fibre can be used for the input to the microscope and as a connection between the microscope output and the monochromator input. In addition, a diffraction grating with more grooves per millimetre can be used for finer spectral lines. It should be noted that suitable software for the prototype micro-Raman spectrometer, in particular, a graphical user interface (GUI), can be developed to control the hardware components and acquire the pixel information of the detector. This will further increase the flexibility of the measurements.

## Figures and Tables

**Table 1:** Mechanical and optical components used for the micro-Raman spectrometer with variable spectral resolution.

Major Parts	Description	Model
Excitation source	A laser with λ_green_ = 532 nm was used for all sample measurements.	DPSS, DHL-W532-500 mW
Sampling unit	A standard optical microscope was used as a sampling unit with a microscope objective of 80× and NA = 0.7.	AMDSP XUM102
Monochromator unit	For the spectral analysis of the scattered light.	LSMO-6 Spectrum Acquisition System with diffraction grating of 11,000 grooves/mm
**Additional parts**	**Description**	**Model**
Bandpass filter (BPF)	The filter used for the elimination of the sideband lines of the DPSS laser.	Thor Labs FL532-1
Notch filter (NF)	Two filters were used to eliminate the stray light mainly produced by intensive Rayleigh elastic scattering positioned at the exit of the microscope.	Thor Labs NF533-17
Focusing lens	Lens used to focus incoming scattered light from the microscope onto the slit of the monochromator.	Solagon 1:1.2/25 mm
Zoom lens	The zoom lens that enabled variable spectral resolution, positioned instead of a second collimating mirror.	Fujinon Lynx II A 18 × 8.5, 1:1.7/8.5–153 mm, 2× Barlow
CCD detector	Used for the detection of scattered light.	Trius Pro 694 Monochromatic
Shutter	Used for the acquisition of dark frame.	Thor Labs SHB05

**Table 2:** Materials used for Raman spectra measurements for the micro-Raman spectrometer prototype with variable spectral resolution.

Material		Measurement	Form
Si	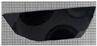	Raman with variable ZF	solid-state
S	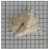	Raman with variable ZF	powder
TiO_2_/P25	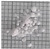	Raman	powder
TiO_2_/800°, 1 h	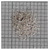	Raman	powder

**Table 3:** Gaussian fit parameters and FWHM values for obtained Si and S spectral peaks.

Si								
Curves	MIN ZF				MAX ZF			
	A	b [cm^−1^]	σ	FWHM	A	b [cm^−1^]	σ	FWHM
P1	1967.66	−526.66	41.20	97.02	7636.75	−518.7	5.67	13.35
P2	2372.75	334.23	70.51	166.05	n/a	n/a		
P3	20,646.45	524.01	27.01	63.61	72,894.81	520.24	5.44	12.81
**S**								
**Curves**	**MIN ZF**				**MAX ZF**			
	**A**	**b [cm^−1^]**	**σ**	**FWHM**	**A**	**b [cm^−1^]**	**σ**	**FWHM**
P1	3073.43	−479.84	37.99	89.48	9557.08	−474.18	7.55	17.8
P2	n/a	n/a			2308.23	−439.01	10.33	24.30
P3	n/a	n/a			3064.33	−257.13	7.26	17.11
P4	5439.25	−247.75	34.02	80.11	22768.3	−229.7	6.08	14.33
P5	n/a	n/a			2932.75	208.63	6.69	15.74
P6	3566.65	250.08	39.2	92.28	6432.75	236.03	12.07	28.43
P7	n/a	n/a			9199.62	433.24	10.51	24.76
P8	16,268.6	475.94	32.68	76.97	49,008.5	472.44	7.76	18.25

**Table 4:** Calculated ANOVA analysis for Raman peaks obtained with two systems using an alpha value of 0.05.

Interval [cm^−1^]	Material	*SS*	*df*	*MS*	*F*	*p*-Value	*F*_critical_
**Si**							
450–600	Between groups	63.43	1	63.43	0.035	0.86	3.86
	Within groups	1,580,470	852	1855.02			
	Total	1,580,533	853				
**S**							
400–550	Between groups	5.79	1	5.79	0.003	0.96	3.86
	Within groups	1,623,253	862	1889.70			
	Total	1,623,259	863				
**TiO_2_ P25**							
350–800	Between groups	9096.03	1	9096.03	0.44	0.51	3.85
	Within groups	60,507,703	2899	20,871.92			
	Total	60,516,799	2900				
**TiO_2_–800°**							
350–800	Between groups	5382.30	1	5382.30	0.32	0.58	3.85
	Within groups	44,032,882	2603	16,916.21			
	Total	44,038,264	2604				

**Table 5:** Calculated parameter for SNR evaluation for micro-Raman spectrometer and reference system.

Material	System	Max Peak [arb. units]	σ_b_ [arb. units]	SNR
Si	Reference	6056.7	9.5831	632.0173
	Prototype	4281.1	22.0753	193.9335
S	Reference	58,326	37.0754	1573.2
	Prototype	106,940	129.4070	826.3716

## Data Availability

The data presented in this study are available upon request from the corresponding author.
